# Psychological Well-Being in Chinese College Students During the COVID-19 Pandemic: Roles of Resilience and Environmental Stress

**DOI:** 10.3389/fpsyg.2021.671553

**Published:** 2021-05-28

**Authors:** Yuanfa Tan, Chienchung Huang, Yun Geng, Shannon P. Cheung, Shuyan Zhang

**Affiliations:** ^1^Research Institute of Social Development, Southwestern University of Finance and Economics, Chengdu, China; ^2^School of Social Work, Rutgers, The State University of New Jersey, New Brunswick, NJ, United States; ^3^School of Government, Central University of Finance and Economics, Beijing, China; ^4^School of International and Public Affairs, Jilin University, Changchun, China

**Keywords:** psychological well-being, resilience, environment stress, students, COVID-19, China

## Abstract

Psychological well-being is an important indicator of well-being and has been found to be associated with a multitude of positive life outcomes. Using data collected from 1,871 Chinese college students from September 23 to October 5, 2020, this study examined students' psychological well-being during the COVID-19 pandemic and investigated how resilience and pandemic-related environmental stress may affect psychological well-being. Results showed that resilience had strong positive effects on psychological well-being during the pandemic. Meanwhile, environmental stress had a moderate effect and marginally reduced psychological well-being. The magnitudes of the estimates suggested that increasing resilience can effectively buffer the negative effect of environmental stress on psychological well-being.

## Introduction

The outbreak of a novel coronavirus disease (COVID-19) has caused serious health threats around the world (Ali et al., 2020). The World Health Organization (WHO) announced that the COVID-19 outbreak could be characterized as a pandemic on March 11, 2020 (WHO, 2020). Empirical evidence has shown the negative effects of the pandemic on the psychological and mental well-being of the general population across several countries (Castelli et al., 2020; Solomou and Constantinidou, 2020; Wang et al., 2020; Xiong et al., 2020). Studies have also focused on the well-being of more specific groups of people, including health care workers (Di Tella et al., 2020; Pappa et al., 2020; Tan et al., 2020) and college students across the globe (Cao et al., 2020; Li et al., 2020; Ma et al., 2020; Wang and Zhao, 2020; Romeo et al., 2021). Wang et al. (2020) conducted an online general population survey between January 31 and February 2 in 2020 to investigate psychological well-being during the initial stages of the COVID-19 outbreak in China. The sample included 1,210 individuals from 194 cities in China. About 54% of the sample stated the psychological effect of the outbreak on them was moderate or severe. Seventeen percentage of the sample experienced moderate to severe depressive symptoms, and 29% of them had moderate to severe anxiety symptoms. Females, students, and individuals with poor health reported higher levels of anxiety and depression (Wang et al., 2020). Likewise, Castelli et al. (2020) utilized an anonymous online survey from March 19 to April 5, 2020 in Italy and found that 20% of the 1,321 participants reported significant post-traumatic stress symptoms. Meanwhile, 69% experienced clinically significant anxiety symptoms, and 31% of the participants experienced clinically significant depressive symptoms. The toll of the pandemic on mental health appears to have been especially heavy for health care workers [see Pappa et al. (2020) for a review], particularly those who work with COVID-19 patients (Di Tella et al., 2020).

Evidence has suggested that university students have higher levels of anxiety and depressive symptoms than general workers during the pandemic (Romeo et al., 2021). In particular, medical students have reported greater mental health problems than the general student population (Cao et al., 2020; Ma et al., 2020). In a February 2020 online survey of Chinese college students from 108 colleges (*N* = 746,217), about 35, 21, and 11% of students reported probable acute stress, depression, and anxiety, respectively (Ma et al., 2020). Ma et al. (2020) also found that epidemic severity in students' respective provinces (measured by cumulative cases of each province prior to March 2020) was positively associated with depression and anxiety. Similarly, in a sample of 7,143 Changzhi Medical College students, Cao et al. (2020) found that about 25% of the students experienced mild or moderate anxiety. Protective factors against anxiety included urban residence, stable family income, and cohabitation with parents. However, having relatives or friends infected with COVID-19 was a risk factor for anxiety. The results also showed that social support reduced level of anxiety (Cao et al., 2020). Likewise, Li et al. (2020) surveyed undergraduate students (*N* = 555) from Hebei Agricultural University between December 2019 and February 2020, finding that students exhibited increased negative affect and symptoms of anxiety and depression after 2 weeks of shelter-in-place during the month of February (Li et al., 2020). Finally, Wang and Zhao (2020) examined anxiety symptoms within a sample of 3,611 Chinese university students just prior to the start of their 2020 spring term. Their anxiety score was significantly higher than the national norm (40.5 vs. 29.8, *p* ≤ 0.001), with about 15% of students meeting the cutoff point of 50 points for a positive anxiety screening.

The pandemic's negative effects on mental health are evident, but current scholarship paints a relatively less clear image of the potential protective factors that may bolster psychological well-being during this challenging time, especially for college students. Understanding the experience of college students during a global pandemic is crucial, given that the college years have been indicated as a critical period for life development (Long, 2008; Li et al., 2012; Costa et al., 2013; Marginson, 2017). Previous research has focused on the manifestation of psychological well-being through anxiety and depression, rather than positive definitions and protective factors. That is, existing studies in the context of the COVID-19 pandemic examine only the absence of illness instead of the presence of wellness. Thus, research has overlooked human capacity, factors that contribute to our thriving, and protective elements associated with well-being (Ryff and Singer, 1996; Ryff, 2014).

Ryff and Singer (1996) proposed that positive psychological well-being contains multiple dimensions, including self-acceptance, positive relations with others, autonomy, environmental mastery, purpose in life, and personal growth. The 6-dimension conceptualization of psychological well-being has been utilized as a comprehensive indicator of psychological functioning and life outcomes in various studies [for a review, see Ryff (2014)]. The integration of mental health and life-span development theories points to the converging aspects of positive psychological functioning (Ryff and Singer, 1996). Studies have shown that life events and experiences, such as relocation (Bardi and Ryff, 2007) and parental death (Maier and Lachman, 2000), are associated with psychological well-being. In addition, psychological well-being is also a good indicator of negative mental health, such as depression (Keyes, 2005), and future life outcomes (Boyle et al., 2009, 2010).

Resilience, or the ability to adapt to various stressors (Wagnild and Young, 1993; Connor and Davidson, 2003), allows individuals to positively cope with adversity and encourages positive adjustment and development in the midst of challenging circumstances (Rutter, 2006; Bajaj and Pande, 2016). Studies have found that high levels of resilience can improve mental health and psychological well-being (Millear et al., 2008; Liossis et al., 2009; Dray et al., 2017). For example, studies on resilience interventions have pointed to the relation between increasing resilience and reducing mental health issues like depressive symptoms, internalizing and externalizing problems, and psychological distress [See Dray et al. (2017) for meta-analysis; Millear et al. (2008)].

Finally, environmental stress and context have been shown to have profound effects on mental health and psychological well-being (Downey and van Willigen, 2005; Gong et al., 2016; Gatersleben and Griffin, 2017; van den Bosch and Meyer-Lindenberg, 2019). For example residential proximity to industrial activity (Downey and van Willigen, 2005) and other physical stimuli and stressors (van den Bosch and Meyer-Lindenberg, 2019) can negatively affect mental health, such as increasing risk of depression (van den Bosch and Meyer-Lindenberg, 2019). Further, studies have shown that exposure to natural disasters (e.g., earthquakes and tsunamis) is associated with poor mental health and increased suicide rates (Kõlves et al., 2013; Musa et al., 2014; Ando et al., 2017). There is also growing evidence of the COVID-19 pandemic's harmful effects on individual psychological and mental well-being (Solomou and Constantinidou, 2020; Wang et al., 2020). Thus, as confirmed COVID-19 cases are reported in the city- and province- levels, local residents may experience heightened environmental risks (i.e., COVID-19 exposure) and greater environmental stress.

Taken together, exposure to environmental stress caused by COVID-19 at the community- and city- levels is likely to affect individual psychological well-being; however, given that resilience may act as a protect factor that promotes psychological well-being, we sought to examine these three variables in relation to one another.

This paper aims to examine the extent of positive psychological well-being in Chinese college social sciences students during the COVID-19 pandemic and to investigate how resilience and environmental stress caused by COVID-19 affect psychological well-being. Based on the above conceptual framework and existing literature, we hypothesize that:

*Hypothesis 1 (H1): resilience was positively associated with psychological well-being of Chinese college students*.

*Hypothesis 2 (H2): environmental stress, caused by COVID-19 pandemic, was negatively associated with psychological well-being of Chinese college students*.

## Materials and Methods

### Data and Sample

An online anonymous survey from junior and senior students in China was utilized to collect the data for this study. Twelve universities located across north, east, south, west and middle regions of China, were selected to have a diverse sample. To be included in this study, participants had to (a) be in their junior or senior year of college and (b) be a social science major. Once universities were chosen, we contacted each university's department of social science and invited junior and senior students to participate in the online survey. We limited our sample to junior and senior students in order to assess the full extent of COVID-19 exposure on college students. Students in the sample had all experienced at least 1 year of college in the past, prior to the onset of the COVID-19 pandemic; they also all experienced interruptions related to the pandemic. As a result, we were able to investigate the level of psychological well-being among students who had experienced college pre-COVID-19, as well as whether resilience increased students' psychological well-being during the COVID-19 pandemic in China. A total of 2,229 students were invited to participate on September 23, 2020, and reminders for invited students were sent 3 and 7 days later. Thousand eight hundred and eighty one students participated in the online survey by October 5, 2020. We excluded the data of ten students due to incomplete survey answers, leaving a final analytical sample of 1,871 students. The response rate was 80%. The research protocol was approved by the research review committee at one of the co-authors' university. An informed consent process was implemented prior to the survey. Students were informed of their voluntary participation and their ability to discontinue survey participation at any time.

### Measures

Psychological well-being was measured by the shortened version of the Psychological Well-being Scale (Ryff and Keyes, 1995). The 18-item scale is broken down into six facets. The first facet, autonomy, refers to self-determination and independence, including the ability to resist social pressures around certain ways of thinking and behaving. Environmental mastery refers to the ability to manage the environment, choosing or creating contexts that are congruent with personal needs and values. The facet of personal growth measures the extent to which an individual feels that they are engaging in continued development. Positive relations refers to the perception that one has warm and trusting interpersonal relationships. Next, purpose in life refers to being goal-driven or being guided by a sense of direction in life. The final facet, self-acceptance, is defined as having a positive attitude toward the self. This involves having self-acceptance and positive feelings about past experiences (Ryff and Keyes, 1995). Each facet was measured by three items. Respondents rated how strongly they agreed or disagreed with corresponding statements using a 7-point scale, in which 1 indicated “strongly agree” and 7 indicated “strongly disagree.” Item responses were reverse-coded so that greater scores indicated greater psychological well-being. The scores of the subscales and the total score were calculated by summing up the corresponding items. Each subscale ranged 3–21, while the score of the whole scale ranged 18–126. The 18-item scale has been used in Chinese population in previous studies with reliability above 0.86 (Wang and Kanungo, 2004; Xu et al., 2020). In our study, the Cronbach's alpha was 0.88.

Two key independent variables in this study were resilience and environmental stress caused by COVID-19 pandemic. Resilience was measured by a shortened version of the Resilience Scale by Wagnild and Young (1993). The 14-item Resilience Scale (RS-14; Wagnild, 2016) assesses resilience-related traits that have been shown to mitigate harmful effects of adverse life circumstances on psychological adjustment (Wagnild and Young, 1993). Past studies have provided evidence of RS-14's cross-ethnic validity among different populations in the U.S., as well as reliability among Chinese adolescents (Pritzker and Minter, 2014; Shi et al., 2016). Participants were prompted to consider the degree to which each item in RS-14 described their experiences over the past 4 weeks. Possible responses ranged from 1 to 7, or strongly disagree to strongly agree, respectively. To calculate the resilience scores, all item responses were added together. Total scores ranged 14–19, with higher scores representing greater spot-measurement of resilience. The Cronbach's alpha for RS-14 for our study sample was 0.91.

Environmental stress caused by the COVID-19 pandemic was measured by the cumulative number of confirmed COVID-19 cases by province. We assigned the cumulative confirmed case number to all students in the same college based on the province in which the college was located, as community- and city-level indicators did not exist in China. Confirmed COVID-19 cases at the province level may capture the extent of COVID-19 disease-related exposure risks and stress faced by the students (Ma et al., 2020). Province-level COVID-19 confirmed cases were retrieved from Caixin (2020) and included confirmed cases up to September 15, 2020.

Socioeconomic characteristics of the respondents acted as control variables in this study. These characteristics included the students' age, gender (0 = male; 1 = female), ethnicity (1 = Han; 0 = other), and household registration (i.e., rural, city, or city with prior rural registration). Information regarding participants' family socioeconomic characteristics was collected as well. These included parents' marital status (i.e., married, separated, divorced, and widowed), parents' highest educational attainment (i.e., elementary school or below, middle school, high school, and some college or above), number of family members, annual family income, and whether their family had received welfare in the last year (0 = no; 1 = yes).

### Analytical Strategy

To examine the distribution of our main variables, we conducted descriptive analysis. This was followed by ordinary least squares (OLS) regression analysis, which allowed us to approximate the net effects of our main independent variables on our dependent variable, while also accounting for the students' socioeconomic characteristics. In this study, we hypothesized that the students' psychological well-being would be determined by their resilience, COVID-19 infection among family and friends, and their socioeconomic characteristics. Both descriptive analysis and OLS regression analysis were performed using STATA statistical software 16.0.

## Results

### Descriptive Statistics

Table 1 presents the descriptive statistics for the main variables. The average score of psychological well-being was 81.7 with a standard deviation (SD) of 12.3. Scores ranged from 24 to 121. Overall, the psychological well-being of the students was high. Among the six subscales of psychological well-being, the highest score was personal growth (14.2), followed by positive relations with others (13.9), environmental mastery (13.8), purpose of life (13.5), autonomy (12.9), and self-acceptance (12.7). The average resilience score was 68.6 with a SD of 13.4. and a range of 14–98. The average COVID-19 confirmed cases in students' provinces was 14,264 (SD = 26,894), ranging from 157 to 68,139. The large range and high standard deviation of this variable together suggest that students resided in provinces with quite different levels of COVID-19 cases. The variance of COVID-19 cases at the province level allows us to examine whether local COVID-19 infection rates affected students' psychological well-being during the pandemic.

**Table 1:** Descriptive statistics of key variables.

	**%**	**Mean (S.D.)**
Psychological Well-being [24–121]		81.7 (12.3)
Autonomy [3–21]		12.9 (2.7)
Environmental Mastery [3–21]		13.8 (2.7)
Personal Growth [3–21]		14.2 (2.5)
Positive Relations with Others [3–21]		13.9 (3.1)
Purpose in Life [3–21]		13.5 (2.6)
Self-acceptance [3–21]		12.7 (2.9)
Resilience [14–98]		68.6 (13.4)
Number of COVID-19 Cases in Province		14,264 (26,894)
Gender [%]		
Female	67.0	
Male	33.0	
Age		20.6 (1.0)
Household Registration [%]		
Rural	38.7	
City, rural before	8.9	
City	52.4	
Grade [%]		
Junior	60.7	
Senior	39.3	
Ethnicity [%]		
Han	89.4	
Others	10.6	
Parent Marital Status [%]		
Married	89.0	
Separated	0.8	
Divorced	6.9	
Widowed	2.4	
Others	0.9	
Parent Highest Education Achievement [%]		
Elementary School and Below	6.9	
Junior High School	28.1	
High School	25.2	
College and above	39.8	
Family Income		90,990 (122,030)
Welfare Status		
No	74.7	
Yes	25.3	
Number of Family Members		3.9 (1.2)

*N = 1,871*.

Table 1 also presents the descriptive statistics of individual socio-demographic characteristics. Majority of the sample was female, mirroring the social science student population in China. The mean age of the sample was 20.6 (SD = 1.0). Over half of the students (52.4%) had city household registration (HR), followed by 38.7% with rural HR, and 8.9% with city but prior rural HR. Majority of students reported that their parents were married (89.0%). The average family income was 90,990 RMB (about 13,580 USD) in the past year, with a standard deviation of 122,030 RMB (18,170 USD). About 25% of students reported that their families received at least one form of social welfare, such as low-income assistance, food subsidies, and other subsidies, in the past year.

### Multivariate Analyses

Table 2 presents the OLS regression standardized estimates of psychological well-being. Given the wide range of COVID-19 cases by province, the variable was first transformed into a natural log number, then entered into regression analyses. The results indicated that resilience and HR had significant effects on psychological well-being. Increasing one standard deviation of resilience led to a 0.51-SD increase in psychological well-being. The finding supports our first hypothesis. Compared to students with rural HR, students with city HR had greater psychological well-being (ß = 0.06). Province-level COVID-19 cases and family income also showed marginal effects on the psychological well-being. A one-SD increase in family income was associated with a 0.03-SD increase in psychological well-being, while the estimated effect of COVID-19 cases on psychological well-being was −0.03. This finding marginally supports our second hypothesis. It is evident that resilience had a large effect on the degree of psychological well-being among the students, while HR, family income, and the number of COVID-19 cases had modest effects on their well-being.

**Table 2:** Regression analysis of psychological well-being.

	**β**	**S. E**.	***P***
Resilience	0.51	0.02	^***^
ln (# of Province COVID-19 Cases)	−0.03	0.13	+
Female	0.03	0.53	
Age	0.00	0.31	
Household Registration: City, rural before	0.01	0.90	
Household Registration: City	0.06	0.66	^*^
Junior	−0.01	0.58	
Han	0.01	0.79	
Married	0.03	0.78	
Junior High School	−0.06	1.04	
High School	−0.03	1.10	
College and above	−0.01	1.16	
Family Income	0.04	0.23	+
Welfare Status	0.03	0.62	
Number of Family Members	−0.03	0.23	
Adjusted R-square	0.29		

*N = 1,871*.

+ *p < 0.10;*

* *p < 0.05*,

*** *p < 0.001*.

We conducted robustness tests on the six subscales of psychological well-being. The regression analyses were conducted similarly to the analysis that produced the results of Table 2, but the dependent variable was replaced by each of the six subscales of the psychological well-being measure. The results are presented in Table 3. For simplicity, we only present the standardized estimates of our key variables, resilience and number of confirmed COVID-19 cases in this table. The results for other variables have been made available in the supplementary materials. Resilience shows significant and positive effects on all six subscales, while confirmed COVID-19 cases shows significant negative effects on personal growth and marginally negative effects on purpose of life. A one-SD increase in resilience was associated the following: 0.48-SD increase in self-acceptance, 0.47-SD increase in environmental mastery, 0.34-SD increase in positive relations with others, 0.33-SD increase in personal growth, 0.31-SD increase in autonomy, and 0.29-SD increase in purpose of life. Students at colleges where confirmed local COVID-19 cases were one SD higher than average had 0.06 SD less personal growth scores, as well as 0.04 SD less purpose in life scores.

**Table 3:** Regression analysis of psychologic well-being subscales.

	**Autonomy**			**Environmental mastery**		
	**β**	**S. E**.	***P***	**β**	**S. E**.	***P***
Resilience	0.31	0.01	^***^	0.47	0.01	^***^
ln (# of Province COVID-19 Cases)	−0.03	0.03		−0.02	0.03	
	**Personal growth**			**Positive relations**		
	**β**	**S. E**.	***P***	**β**	**S. E**.	***P***
Resilience	0.33	0.01	^***^	0.34	0.01	^***^
ln (# of Province COVID-19 Cases)	−0.06	0.03	^**^	−0.03	0.04	
	**Purpose in life**			**Self-Acceptance**		
	**β**	**S. E**.	***P***	**β**	**S. E**.	***P***
Resilience	0.29	0.01	^***^	0.48	0.01	^***^
ln (# of Province COVID-19 Cases)	−0.04	0.03	+	0.01	0.03	

*N = 1,871*.

+ *p < 0.10;*

** *p < 0.01*,

*** *p < 0.001*.

## Discussion

Empirical evidence has shown that the pandemic poses negative effects on the psychological and mental well-being of various populations across countries (Cao et al., 2020; Castelli et al., 2020; Di Tella et al., 2020; Pappa et al., 2020; Solomou and Constantinidou, 2020; Xiong et al., 2020). Less is known about the effects of the pandemic with regards to positive dimensions of health, such as psychological well-being, as well as with regards to the protective factors that bolster psychological well-being. We sought to examine the psychological well-being of college students given the time spent in college is a critical developmental period (Long, 2008; Costa et al., 2013; Marginson, 2017). Although it is important to identify factors associated with mental health problems such as anxiety, studies on factors increasing the presence of wellness are important to better understand humans' capacity to thrive in adverse conditions and circumstances (Ryff and Singer, 1996; Ryff, 2014).

The findings of this study, unlike previous studies from the early stages of the pandemic (Cao et al., 2020; Li et al., 2020; Ma et al., 2020; Wang et al., 2020; Romeo et al., 2021), show that a majority of Chinese students had good overall psychological well-being in September 2020. This difference may be attributed to the actions taken to control the COVID-19 outbreak in China. As the rate of confirmed cases slowed down, China's government gradually removed lockdown measures for many cities in March 2020. The first and last city to enter and lift lockdown, Wuhan, removed lockdown measures on April 8, 2020. Confirmed COVID-19 cases in China have been kept stable at around 90,000 cases since the removal of these lockdown measures (Caixin, 2020). In September 2020, a majority of colleges in China resumed the academic school year, including in-person teaching on most campuses (Nierenberg and Pasick, 2020); in all, life in China has begun to look the way that it did pre-COVID-19 (Hernández, 2020).

The students in this study scored relatively high in the psychological well-being subdimensions of personal growth, positive relations with others, environmental mastery, and purpose in life. It is worthy to note that autonomy and self-acceptance scores were relatively lower than other scores. Overall, students in the survey expressed relatively high feelings related to continued development but less so on possessing a positive attitude toward the self during the pandemic. Lower scores for positive attitudes toward the self might be attributed to students' perceived lack of control during the pandemic. Positive self-regard, self-esteem, and self-perception have been found to be greater among adolescents with an internal locus of control (LOC) (Cazan and Dumitrescu, 2016). LOC refers to the extent to which an individual believes their outcomes are affected by their own actions (Rotter, 1990). Those with external LOC believe their own actions have little to no effect on outcomes, while those with internal LOC believe that they are able to control their outcomes. It is possible that during the pandemic, students felt a loss of control, which subsequently led to a decrease in positive self-regard.

With respect to the two key independent variables examined in this study, a majority of students had a high level of resilience. Since we purposely sampled students across China, there was great variance in the numbers of confirmed COVID-19 cases by province. Some provinces had only 157 confirmed cases (e.g., Jilin Province), while others reported over 68,000 confirmed cases (e.g., Hubei Province). This variance allowed us to examine the extent to which environmental stress caused by COVID-19 cases affects the living environment and, subsequently, the psychological well-being of students during the pandemic.

The findings from the regression analyses indicate strong and robust effects of resilience on psychological well-being during the pandemic. Resilience significantly increased overall psychological well-being scores, as well as well-being as it pertains to each of the six dimensions. This is consistent with existing studies that have found that resilience positively predicts psychological well-being specifically among students in higher education (Souri and Hasanirad, 2011; Malkoc and Yalcin, 2015). Among Chinese student populations, a positive relation between resilience and psychological well-being has been found in undergraduate nursing students specifically (Smith and Yang, 2017). Our study adds to the existing literature by contextualizing the importance of this relation during a collective trauma (i.e., the COVID-19 pandemic) within a more representative student sample in China.

Our results also indicate that environmental stress has an effect on psychological well-being to some degree. Province-level COVID-19 cases had marginal effects on overall psychological well-being. The number of confirmed province-level COVID-19 cases was negatively associated with overall psychological well-being. The number of confirmed COVID-19 cases appeared to have stronger effects on personal growth and purpose in life. Our finding that environmental stress only marginally affected psychological well-being may be due to the fact that COVID-19 confirmed cases were measured at the province level, rather than at the city- or community-levels. The community and city environment indicators may be better measures of environmental stress since they represent students' more immediate environments; however, due to data limitations, we were unable to control for confirmed COVID-19 cases at more local levels.

Combining the findings from overall psychological well-being and the subscales of psychological well-being, our study suggests that resilience and environmental stress indeed affect students' well-being during the pandemic. These findings offer practical implications for mental health providers. The magnitudes of the estimates suggest that increasing resilience can effectively improve psychological well-being and buffer the negative effects of environmental stress on psychological well-being during the COVID-19 pandemic. Thus, programs that increase student resilience can also improve their psychological well-being during the pandemic. Recent studies have shown that several programs, including mindfulness, mental awareness, and life skills programs, can positively affect students' resilience (Bajaj and Pande, 2016; Galante et al., 2018; Lu et al., 2018; Huang et al., 2019). Thus, colleges may seek to offer their student communities opportunities for mindfulness-related training and life skills programs during the pandemic.

Finally, the findings of this study also revealed that several socioeconomic characteristics, such as low family income and rural HR, are related to low psychological well-being. This is consistent with previous studies that have indicated that individuals with low socioeconomic characteristics had higher health risk perception compared to their counterparts (Commodari et al., 2020). Additionally, this is consistent with more recent findings that have emphasized that these already-vulnerable populations experience heightened vulnerability during the COVID-19 pandemic (Douglas et al., 2020; Rudenstine et al., 2021). In particular, U.S.-based low-income college students appear to experience high prevalence of both anxiety and depression symptoms (Rudenstine et al., 2021). In our sample, students with rural HR may be disadvantaged similarly, as they typically have low socioeconomic characteristics compared to students with city HR. As such, these students may have lower psychological well-being due to perceived lack of supports. Thus, interventions and services to improve student psychological well-being may require targeted outreach to high-risk subgroups. In the Chinese university context, this includes low-income students and students with rural HR.

This study has several limitations that may provide research implications for future studies. First, analysis using a cross-sectional dataset may only approximate associative relations among variables. Thus, we are unable to infer causal relations among our main variables—resilience, environmental stress, and psychological well-being during the COVID-19 pandemic. A longitudinal study design may better approximate such relations. Next, this study is subject to omitted variable bias, considering that unobserved variables (e.g., peer support and academic stressors) may have affected psychological well-being within the student sample. Third, we used measurements that relied on self-reporting to assess for resilience and psychological well-being. Issues with self-reporting include both unintended and intended reporting errors. For example, in the case of social desirability bias, students may be compelled to over-report their psychological well-being due to stigma related to poor mental health. To address this, future studies can use data triangulation by gathering information from peer and teacher reports to verify the findings of this study. Additionally, although the study sample size and diversity of colleges from which we drew our sample increase our confidence in the results, the generalizability of these findings to the larger college population in China is unknown and requires further research. Fifth, the small effects of province-level COVID-19 cases may be a result of measurement errors between smaller communities and the province itself. Future research using community indicators of environmental stress is warranted to examine the effects of immediate environmental stress on psychological well-being.

## Conclusion

With data collected from 1,871 college students across China, we investigated the degree of positive psychological well-being in college students studying social science during the COVID-19 pandemic. We examined how resilience and environmental stress may affect the students' psychological well-being. Results of OLS regression analysis show that during the pandemic, resilience had a strong positive relation with psychological well-being, while environmental stress marginally reduced psychological well-being. Despite the limitations that we have discussed, the present study contributes to existing knowledge on the factors that may contribute to the psychological well-being of Chinese students during the COVID-19 pandemic.

## Data Availability Statement

The raw data supporting the conclusions of this article will be made available by the authors, without undue reservation.

## Ethics Statement

The studies involving human participants were reviewed and approved by Rutgers University IRB. Written informed consent for participation was not required for this study in accordance with the national legislation and the institutional requirements.

## Author Contributions

YT, CH, and YG: conceptualization and resources. YT and CH: methodology and software. CH, SC, and SZ: validation. YT, CH, YG, SC, and SZ: formal analysis and writing—original draft preparation. YT, CH, YG, and SZ: investigation and data curation. All authors contributed to the article and approved the submitted version.

## Conflict of Interest

The authors declare that the research was conducted in the absence of any commercial or financial relationships that could be construed as a potential conflict of interest.
