# Supplementary material for: Psychological Well-Being in Chinese College Students During the COVID-19 Pandemic: Roles of Resilience and Environmental Stress
Source: Front Psychol. 2021 May 28;12:671553. doi: 10.3389/fpsyg.2021.671553 (PMC8192835; doi:10.3389/fpsyg.2021.671553)
Supplement: Supplementary file 1 [file Table_1.docx]

Supplementary Material: Regression Results of Control Variables in Psychological Well-being Subscale Analyses

|  | **Autonomy** | | | **Environmental Mastery** | | | **Personal Growth** | | |
| --- | --- | --- | --- | --- | --- | --- | --- | --- | --- |
|  | **β** | **S. E.** | **P** | **β** | **S. E.** | **P** | **β** | **S. E.** | **P** |
| Female | -0.11 | 0.13 | *** | 0.03 | 0.12 |  | 0.07 | 0.12 | ** |
| Age | -0.02 | 0.08 |  | 0.01 | 0.07 |  | -0.03 | 0.07 |  |
| Household Registration: City, rural before | 0.02 | 0.22 |  | 0.01 | 0.21 |  | 0.00 | 0.20 |  |
| Household Registration: City | 0.05 | 0.16 | + | 0.04 | 0.15 |  | 0.09 | 0.15 | ** |
| Junior | 0.03 | 0.15 |  | -0.01 | 0.14 |  | -0.02 | 0.13 |  |
| Han | 0.00 | 0.20 |  | -0.01 | 0.18 |  | -0.02 | 0.18 |  |
| Married | 0.01 | 0.19 |  | 0.02 | 0.18 |  | 0.01 | 0.17 |  |
| Junior High School | -0.05 | 0.26 |  | 0.00 | 0.24 |  | -0.08 | 0.23 | + |
| High School | -0.02 | 0.27 |  | 0.01 | 0.25 |  | -0.07 | 0.24 |  |
| College and above | 0.00 | 0.29 |  | 0.01 | 0.27 |  | -0.07 | 0.26 |  |
| Family Income | -0.02 | 0.06 |  | 0.03 | 0.05 |  | 0.03 | 0.05 |  |
| Welfare Status | -0.01 | 0.15 |  | 0.03 | 0.14 |  | 0.05 | 0.14 | + |
| Number of Family Members | -0.03 | 0.06 |  | -0.02 | 0.05 |  | -0.03 | 0.05 |  |

Note: N=1,871. + p<.10; * p < .05, ** p <.01, *** p < .001.

Supplementary Material: Regression Results of Control Variables in Psychological Well-being Subscale Analyses (Continued)

|  | **Positive Relations** | | | **Purpose in Life** | | | **Self-acceptance** | | |
| --- | --- | --- | --- | --- | --- | --- | --- | --- | --- |
|  | **β** | **S. E.** | **P** | **β** | **S. E.** | **P** | **β** | **S. E.** | **P** |
| Female | 0.07 | 0.15 | ** | 0.07 | 0.13 | ** | 0.00 | 0.15 |  |
| Age | 0.03 | 0.08 |  | -0.01 | 0.07 |  | 0.00 | 0.08 |  |
| Household Registration: City, rural before | 0.02 | 0.24 |  | -0.01 | 0.21 |  | -0.01 | 0.25 |  |
| Household Registration: City | 0.02 | 0.18 |  | 0.02 | 0.16 |  | 0.05 | 0.18 | + |
| Junior | -0.05 | 0.16 | + | -0.02 | 0.14 |  | 0.02 | 0.16 |  |
| Han | 0.04 | 0.22 | + | 0.02 | 0.19 |  | 0.02 | 0.22 |  |
| Married | 0.04 | 0.21 | + | 0.01 | 0.18 |  | 0.02 | 0.22 |  |
| Junior High School | -0.07 | 0.29 |  | -0.07 | 0.25 |  | 0.00 | 0.29 |  |
| High School | -0.04 | 0.30 |  | -0.07 | 0.26 |  | 0.03 | 0.30 |  |
| College and above | -0.02 | 0.32 |  | -0.01 | 0.27 |  | 0.03 | 0.32 |  |
| Family Income | 0.05 | 0.06 | * | 0.05 | 0.06 | * | 0.02 | 0.06 |  |
| Welfare Status | 0.04 | 0.17 | + | 0.00 | 0.14 |  | 0.01 | 0.17 |  |
| Number of Family Members | -0.05 | 0.06 | * | -0.02 | 0.05 |  | 0.00 | 0.06 |  |

Note: N=1,871. + p<.10; * p < .05, ** p <.01.
